# Spatial and Genetic Diversity of Clinical Isolates of *Blastocystis* in Italy: A Network Analysis

**DOI:** 10.3390/pathogens14020139

**Published:** 2025-02-03

**Authors:** Isabel Guadano-Procesi, Federica Berrilli, David Di Cave

**Affiliations:** Department of Clinical Sciences and Translational Medicine, Faculty of Medicine, University of “Tor Vergata”, 00133 Rome, Italy; berrilli@uniroma2.it (F.B.); dicave@uniroma2.it (D.D.C.)

**Keywords:** *Blastocystis*, phylogenesis, haplotype

## Abstract

*Blastocystis* is a common intestinal protist with a global distribution, frequently found in humans and various animals. Despite its prevalence, its role in human health remains debated, oscillating between being a harmless commensal and a potential pathogen. It has also been associated with gastrointestinal disorders such as irritable bowel syndrome (IBS) and inflammatory bowel disease (IBD). In Italy, the genetic and spatial diversity of *Blastocystis* remains understudied, despite the country’s diverse urbanized and environmental landscapes. This study investigates the haplotypic and spatial diversity of clinical isolates of *Blastocystis* across two different Italian regions, with an emphasis on subtype distribution and genetic variation. Using a network-based haplotype analysis, the study reveals a heterogeneous subtype distribution, with subtype ST4 (47.3%) being the most prevalent, followed by ST3 (20%), ST1 (16.4%), ST2 (12.7%), ST6 (1.8%) and ST7 (1.8%). The overall infection rate detected from symptomatic patients is 9.75%. Notably, ST4 shows limited haplotypic variation, suggesting a more stable population structure that is potentially linked to a human-adapted lineage. In contrast, ST1 and ST2 exhibit greater haplotypic diversity, likely due to ongoing zoonotic transmission. These findings enhance our understanding of the epidemiology of *Blastocystis* in Italy and underscore the need for further research on its pathogenic potential and transmission dynamics.

## 1. Introduction

*Blastocystis* is a common intestinal Stramenopiles with a cosmopolitan distribution, frequently detected in humans and a wide variety of animal hosts. Despite its widespread occurrence, its role in human health remains contentious, oscillating between commensalism and potential pathogenicity. Numerous studies have associated *Blastocystis* with gastrointestinal symptoms, including irritable bowel syndrome (IBS) and inflammatory bowel disease (IBD), although conclusive evidence regarding its pathogenicity is still lacking [1]. The organism’s genetic diversity and potential zoonotic transmission have been emphasized as crucial elements in understanding its epidemiology [2].

Italy offers a unique setting for the study of *Blastocystis*, given its diverse environmental and urbanized landscapes, which may influence the distribution and genetic structure of *Blastocystis* populations. While several studies have documented the presence of *Blastocystis* in Italian populations, a comprehensive analysis of the spatial and genetic diversity of the protist across different regions remains scarce [3].

Haplotypic analysis provides a powerful approach for understanding intraspecies genetic diversity and its association with host and geographic factors. By focusing on haplotypes—specific genetic variants found within a population—it is possible to trace the evolutionary history of *Blastocystis* strains, infer patterns of transmission, and explore potential connections between genetic variants and pathogenicity. Previous studies have identified more than 40 distinct subtypes of *Blastocystis*, with ST1 to ST4 being the most prevalent in humans worldwide [4]. However, within these subtypes, considerable haplotypic diversity exists [3,5].

Nevertheless, little is known about the haplotypic diversity within these subtypes and how it correlates with spatial factors such as geographic location or urbanization levels. Network-based haplotype analysis can reveal hidden structures within *Blastocystis* populations by clustering isolates with shared genetic features, allowing for a deeper understanding of the spatial relationships and potential transmission routes.

This study aims to characterize the spatial and genetic diversity of *Blastocystis* clinical isolates from Italy, with a particular focus on haplotypic variation. Through network analysis, we will explore the connections between different haplotypes across two Italian regions, highlighting potential epidemiological patterns in shaping *Blastocystis* diversity. By integrating genetic and spatial data, this research attempts to fill the existing knowledge gaps on the distribution and evolution of *Blastocystis* in Italy, contributing to a broader understanding of its public health implications.

## 2. Materials and Methods

### 2.1. Sampling

In this study, 791 fecal samples from 636 symptomatic patients were analyzed. These samples were collected between July 2021 and October 2022 at the Unit of Parasitology of the Azienda Ospedaliera Universitaria Policlinico Tor Vergata (PTV) of Rome, Central Italy, which handles over 50,000 accesses annually. The samples were tested for *Blastocystis* using the Allplex™ Gastrointestinal Panel—Parasite Assay for Protozoa Detection in Stool Samples. Due to the higher sensitivity of RT-PCR, only isolates with cycle threshold (Ct) values lower than 30 were selected. In the case of multiple fecal samples from a single patient testing positive for the parasite, the isolate with the lowest Ct value was used for the amplification and considered only once for calculating the infection rate. Genotyping of a 600 bp segment of the *Blastocystis* SSU-rDNA was performed using end-point PCR as described by Scicluna et al. [6]. Amplicons were purified with the mi-PCR Purification Kit (Metabion International AG, Planegg, Germany) and sent to Bio-Fab Research (Rome, Italy) for sequencing. The chromatograms obtained were manually inspected with Finch TV 1.4 software (Geo-Spiza, Inc., Seattle, WA, USA) to detect any potential double peaks, which could indicate mixed infections or single-nucleotide polymorphisms (SNPs).

For each patient, sex and age data were collected. Diagnostic results were anonymized before analysis, as were patient data prior to processing. The identity of each participant was protected by the attribution of an anonymous code to each stool sample collected. This research was conducted without any “invasive act” affecting the physical, psychological or moral integrity of the participants. All experiments were performed following the ethical standards established by the Universal Declaration of Human Rights (1948) and the Declaration of Helsinki (1964), and its successive amendments were complied with.

The new samples obtained, along with 24 *Blastocystis* sequences from patients attending the same hospital between March 2014 and March 2017, retrieved by the Unit of Parasitology, were collected to build the dataset, namely DATASET1.

### 2.2. Subtype, Allele Attribution and Phylogenetic Analysis

All isolates in DATASET1 were tested using PubMLST [7] to be properly associated with a subtype and an allele. The phylogenetic analysis focused on the sequences present in DATASET1 and was conducted using MEGA11 software [8]. The Neighbor-Joining (NJ) method was employed, based on the Tamura 3-parameter model, as selected by ModelTest using the Akaike Information Criterion (AIC). A bootstrap value of 1000 was applied to assess the robustness of the obtained clades. 

### 2.3. Statistical Analysis

The results obtained from the comparison of subtypes and different alleles of *Blastocystis*, as well as the age of the patients, were evaluated using the chi-square test (χ^2^). A *p*-value < 0.05 was considered statistically significant. All statistical analyses were conducted using IBM SPSS Statistics software [9].

### 2.4. Haplotype and Network Analysis

To facilitate an appropriate comparison between our data and those available in Italy, another dataset was constructed (DATASET2) encompassing all *Blastocystis* sequences present in GenBank from humans as hosts in Italy and also including those collected in DATASET1. In case of ambiguous sequences (unphased data) from DATASET1, the sequences were excluded from the analysis. To obtain the sequences from GenBank, a multistep strategy was employed by entering the keywords “*Blastocystis*” AND/OR “Italy” AND/OR “Italian” into the search field of the “Nucleotide database” and considering only sequences with 99% identity with *Blastocystis* sp. This was followed by a thorough verification of the “Country Isolation and Host” information to ensure the accuracy of the data collected.

The haplotype analysis was conducted on DATASET2 on polymorphic sites using DnaSP v.6 software [10] and Tajima’s D test [11]. PoPART version 1.7 (Population Analysis with Reticulate Trees) genetic software [12] was used to perform the minimum spanning network calculation [13]. The analysis was performed with sequences trimmed to the shortest length with high-quality fragments and sites, considering alignment gaps. 

## 3. Results

In this study, 62 fecal samples from 636 patients tested positive for *Blastocystis* during the analysis period of July 2021–October 2022, with an infection rate of 9.75%. Among them, 37 isolates with a cycle threshold (Ct) of <30 were analyzed. The molecular analysis, conducted using the previously described PCR protocol, yielded high-quality sequences for 31 out of 37 samples. All sequences were identified as *Blastocystis*, demonstrating high identity values ranging from 99.46% to 100% with homologous sequences of *Blastocystis* isolates deposited in GenBank and PubMLST.

### 3.1. Subtype, Allele Attribution and Phylogenetic Analysis

Subsequently, DATASET1 was constructed, incorporating the sequences from the 31 isolates obtained in the period of July 2021–October 2022, along with 24 previously acquired *Blastocystis* sequences (March 2014–March 2017), as detailed in Section 2 (Materials and Methods), resulting in a total of 55 sequences from symptomatic patients. Among these, 32 sequences were from females (58.2%) and 18 were from males (32.7%). For five patients, these data could not be obtained. The ages of the patients ranged from 6 to 88 years, with a mean age of 47.85 years. In Table 1, *Blastocystis* subtypes based on the demographic characteristics of the patients are shown. The statistical analysis suggested that the ST distribution is independent of the age and gender of the patient.

The sequences were analyzed using the PubMLST website to correctly attribute the subtype and associated allele. Each sequence was associated with a single stool sample belonging to a patient. The results obtained for the isolates present in DATASET1 are shown in Table S1.

Overall, 9 sequences were assigned to subtype ST1 (9/55; 16.4%) with 3 isolates (BHHS3, BHHS12, BHHS51) showing identical sequences; 7 to subtype ST2 (7/55; 12.7%) with BHHS19 and BHHS2 equal to BHHS20 and BHHS6, respectively; 11 to subtype ST3 (11/55; 20%) with all identical sequences except for BHHS1 and BHHS23; and 26 to subtype ST4 (26/55; 47.3%), with sequences that were all identical. Additionally, two samples were attributed, one to subtype ST6 (1/55; 1.8%) and the other to subtype ST7 (1/55; 1.8%). Representative sequences within each subtype were deposited in GenBank under the accession numbers PQ483073–PQ483086; PQ565643–PQ565647.

From the analysis of the alleles, eight different variants were identified across the various subtypes (Table 2). A single allele was found for subtype ST1 (allele 4), and similarly, ST4 was particularly homogeneous, represented by a single allele (allele 42). The alleles identified for subtypes ST6 and ST7 were allele 123 and allele 137, respectively. For subtypes ST2 and ST3, two different alleles were observed. In the case of ST2, alleles 9 and 12 were identified. For subtype ST3, two alleles, allele 34 and allele 36, were found, with allele 34 also being more prevalent, accounting for 91% of the samples.

**Figure 1:**
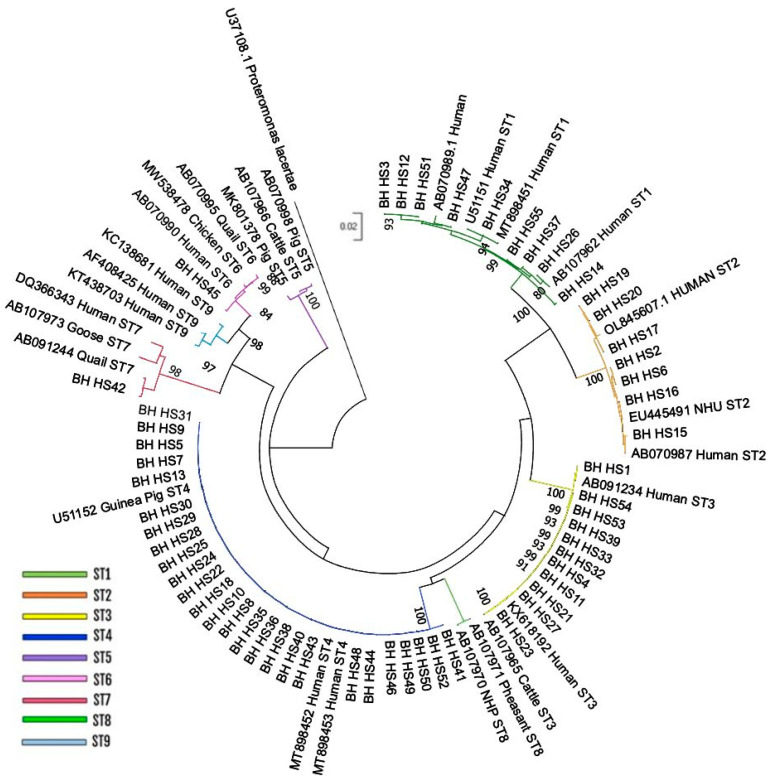
Phylogenetic analysis performed using the Neighbor-Joining (NJ) method on the partial *Blastocystis* SSU-rDNA sequences present in DATASET1, comparing them with reference sequences obtained from GenBank. The values at the nodes of the tree indicate bootstrap values greater than 80%. The clades are color coded to reflect their assignments to different subtypes (STs).

The phylogenetic analysis allowed for the grouping of the isolates in DATASET1 into six distinct subtypes of *Blastocystis*, confirming the results obtained through the PubMLST website. Specifically, of the 55 isolates present in the dataset, 9 clustered with ST1, 7 formed a clade with reference sequences of ST2, 11 grouped with ST3 sequences, and 26 clustered with reference sequences of ST4. Additionally, two isolates corresponded to ST6 and ST7, forming a distinct clade with their respective reference sequences (Figure 1). The topology of the Neighbor-Joining tree generated using the partial SSU-rDNA gene showed two different well-defined clades, formed by ST1/ST2 and ST6/ST7/ST9 and supported by high bootstrap values (100 and 98, respectively), while ST5 relies on a separated branch. Less defined is the relationship between ST3, ST4 and ST8.

### 3.2. Statistical Analysis

No statistically significant correlation was found between the different subtypes and alleles of *Blastocystis* and age/sex of the patients. The chi-square test (χ^2^) results yielded *p*-values greater than 0.05, indicating the absence of any significant association.

### 3.3. Haplotype and Network Analyses

Through the search specified in Section 2 (Materials and Methods), 54 sequences from Italy were obtained from GenBank, allowing the construction of DATASET2, also including those collected in DATASET1 (excluding two ambiguous sequences, thus considering 53 sequences from DATASET1) for a total of 107 sequences. The selected region spanned 561 sites. Alignment gaps were included in the evaluation, and the number of variable sites identified was 166. Regarding haplotype distribution, 46 distinct haplotypes were observed (see Table S2), resulting in a haplotypic diversity (Hd) of 0.8850.

The most frequently observed haplotypes (hp) were hp34 and hp41, associated with ST3 and ST4, respectively. Subtype2, on the other hand, was mostly associated with hp22 and hp20, while ST1 was linked to hp7 and hp5.

The variability observed across different subtypes in terms of haplotypes showed no variability in ST4, which was entirely represented by hp41 and allele 42 (except for one isolate belonging to hp40 and allele 89). Moderate variability was observed in ST3, which was mostly associated with hp34 and allele 34, while ST1 and ST2 exhibited high variability.

**Figure 2:**
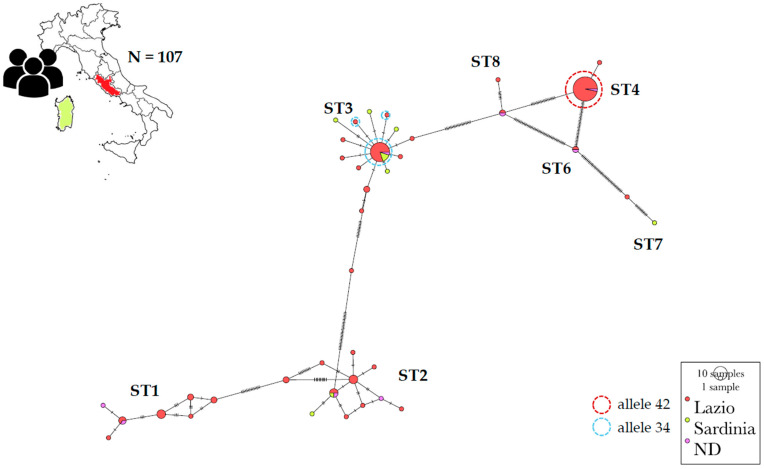
Minimum spanning haplotype networks built in PoPART software for DATASET2 (107 sequences). Haplotypes are represented by circles proportional to relative haplotype abundance; different shades indicate different areas of origin. Notches refer to the mutational steps between haplotypes. Colored circles indicate alleles most represented for the single haplotypes. Subtypes and alleles are indicated. NDs indicates Italian regions not defined in the reference.

From a spatial perspective, the network analysis showed hp41 (ST4) and haplotypes within ST1 to be predominantly associated with the Lazio region, to a lesser extent with other Italian regions (NDs) and absent in Sardinia. In contrast, hp 34 (ST3) and haplotypes related to ST2 were represented in Lazio and Sardinia as well as in several other Italian regions (NDs). Of note is the presence of four specific haplotypes for ST3 and one for ST2 in Sardinia (Figure 2).

## 4. Discussion

This study sheds light on the genetic and spatial diversity of *Blastocysti*s in Italy, adding data for a more comprehensive view of the parasite haplotypic structure across multiple subtypes.

The prevalence of the different subtypes related to the isolates obtained in DATASET1 reveals that ST4 was the most frequently identified, accounting for 47.3% of all samples, followed by ST3 (20%), ST1 (16.4%), ST2 (12.7%), ST6 (1.8%) and ST7 (1.8%). These findings are consistent with trends observed in several European countries where ST4 is frequently reported together with ST3 as the dominant subtypes in human isolates [14]. For instance, in a study conducted in Denmark, ST4 also showed a high prevalence among symptomatic patients, indicating a potential regional clustering of this subtype [15]. Moreover, the predominance of ST4 parallels findings from studies in other southern European countries, where environmental factors (such as habitat overlap and urbanization) and human behavior (for instance, close contact between people and animals or low awareness of prevention methods) likely contribute to its successful establishment in human populations. The distribution of ST4 contrasts between Europe and regions such as West and South Asia, the Americas, and Africa (less frequently detected) [14]. Rodents have been identified as reservoir hosts of ST4 [15], but exposure to rodent feces is likely to be universal. These observations seem to suggest that other environmental, cultural and perhaps host-specific factors could be influencing the geographical distribution of ST4 and, in general, of the other subtypes, though the exact reasons remain uncertain.

The low haplotypic variation observed in all *Blastocystis* sequences available from Italy (DATASET2) in ST4 contrasts with the higher haplotypic variability detected in ST1 and ST2, and with the moderate haplotypic variability of ST3. Subtype ST4 was almost exclusively represented by haplotype hp41 and allele 42 (except for one isolate), whereas ST1 and ST2 demonstrated significant haplotypic heterogeneity. The results presented here align closely with those of Mattiucci et al. [3], particularly regarding the dominance of ST3 and ST4 and the limited haplotypic diversity within this last subtype. The widespread presence of almost a single haplotype, as revealed in this study and as reported in previous work [3], supports the idea of a relatively stable and homogeneous population.

The absence of haplotypic variation within ST4 that supports the hypothesis of a stable and homogeneous population in the human host could be related to a recent evolutionary entry into the human population [3,5,16]. The evolutionary history of *Blastocystis* subtypes could offer an explanation for this lack of diversity in ST4: the variant might have undergone a more recent evolutionary bottleneck, resulting in reduced genetic variation compared to other subtypes.

In contrast, ST1 and ST2, which exhibit significantly higher haplotypic diversity, are thought to have a more complex evolutionary history involving zoonotic transmission [17]. Both subtypes are frequently found in humans as well as various animals, suggesting ongoing genetic exchange between human and animal populations. This continual interspecies transmission likely contributes to the higher haplotypic diversity observed in ST1 and ST2, which show a wide range of haplotypes. These findings suggest that *Blastocystis* subtypes with zoonotic potential are generally more genetically diverse due to their broader host range and exposure to different environmental pressures.

The spatial distribution of haplotypes also provides interesting insights into the epidemiology of *Blastocystis* in Italy. Haplotypes associated with ST4 (hp41) were predominantly found in the Lazio region, while haplotypes associated with ST1 and ST3 were more widely distributed. Of note is the absence of ST1 and ST4 [18] and the presence of single rare haplotypic variants on the island of Sardinia, further emphasizing the complex interaction between environmental factors and *Blastocystis* transmission dynamics. In fact, given the geographical nature of the Sardinia, it could be hypothesized that this genetic setting may be linked to the founder effect: this phenomenon is very common on islands, where speciation processes are particularly intense. However, it is important to note that the spatial analysis conducted in this study is still incomplete due to the scarcity of data from Italy. This limitation introduces a bias that should be addressed through new sequences and further studies on *Blastocystis* from other Italian regions.

The lack of haplotypic variation in ST4 raises intriguing questions about its pathogenic potential. Although *Blastocystis* pathogenicity has remained a topic of debate for many years, more recent research, particularly studies examining *Blastocystis* in the context of gut microbiota, seems to suggest that it is generally linked to a healthy bacterial community [19,20]. Nevertheless, some evidence suggests that a more complex and nuanced perspective may be needed [21]. The genetic homogeneity of ST4 could imply a more stable interaction with the human host. However, considering that, in the present study, only isolates from symptomatic patients were analyzed, further research is needed to explore whether the reduced diversity in ST4 has any direct implications for its virulence or its ability to cause disease in human populations.

## 5. Conclusions

In conclusion: the findings of this study offer new insights into the genetic and spatial characteristics of *Blastocystis* subtypes in Italy with evidence of a possible link between geographical distribution and subtype variability, likely reflecting the specific ecological and anthropogenic contexts of the Italian landscape.

Particular attention was given to the low haplotypic diversity detected in ST4. The evolutionary and epidemiological implications of its reduced haplotype variability suggest that ST4 is a relatively recent and possibly human-adapted lineage, with limited zoonotic transmission potential, making it a key subtype for further investigation in terms of pathogenicity and transmission dynamics. Future research should focus on exploring the evolutionary history of ST4 in more detail, and on investigating whether its genetic stability impacts its role in human health.

Limitations of the study: This study presents some limitations that must be considered for proper interpretation of the results. In particular, the limited number of samples does not provide a comprehensive picture of subtype/allele/haplotype distribution of *Blastocystis* in the country with a lack of diversity due to the low number of regions included (two regions plus some NDs). Moreover, detailed supplementary data (such as specific associations between different STs and symptoms, drug resistance and pathogenicity) will be significant to complement foundational studies, which aim to expand data repositories for a broader understanding of *Blastocystis*. Integrating these elements would significantly enhance the value and impact of baseline studies.

## Figures and Tables

**Table 1:** Subtypes correlated with age classes and gender for DATASET1; data were available for 50 out of 55 patients.

**Age Class**	**ST1**	**ST2**	**ST3**	**ST4**	**ST6**	**ST7**
0–15	-	2	3	2	-	-
16–32	2	-	1	1	-	1
33–49	2	2	4	8	1	-
50–66	1	-	-	3	-	-
67–85	3	2	2	9	-	-
86–99	-	-	-	1	-	-
**Gender**	**ST1**	**ST2**	**ST3**	**ST4**	**ST6**	**ST7**
F	4	4	6	16	1	1
M	4	2	4	8	-	-

**Table 2:** Allele distribution among the subtypes obtained from the 55 sequences present in DATASET1.

Subtype	N of Isolates	Allele Identification
ST1	9	4
ST2	4	9
ST2	3	12
ST3	10	34
ST3	1	36
ST4	26	42
ST6	1	123
ST7	1	137

## Data Availability

The data that support the findings of this study are available in the public database GenBank “https://www.ncbi.nlm.nih.gov/nucleotide/ (accessed 2 February 2025)”.

## Supplementary Materials

The following supporting information can be downloaded at: https://www.mdpi.com/article/10.3390/pathogens14020139/s1. Table S1. DATASET1, with STs and alleles indicated for each isolate; Table S2. DATASET2, with haplotypes distribution.
