# Supplementary material for: Spatial and Genetic Diversity of Clinical Isolates of Blastocystis in Italy: A Network Analysis
Source: Pathogens. 2025 Feb 3;14(2):139. doi: 10.3390/pathogens14020139 (PMC11858726; doi:10.3390/pathogens14020139)
Supplement: Supplementary file 1 [file pathogens-14-00139-s001.zip › pathogens-3343569-supplementary.pdf]

pathogens

MDPI

Supplementary materials:

Table S1. DATASET1, with STs and alleles indicated for each isolate

Isolate	Subtype	Allele
BHHS1	ST3	36
BHHS2	ST2	9
BHHS3	ST1	4
BHHS4	ST3	34
BHHS5	ST4	42
BHHS6	ST2	9
BHHS7	ST4	42
BHHS8	ST4	42
BHHS9	ST4	42
BHHS10	ST4	42
BHHS11	ST3	34
BHHS12	ST1	4
BHHS13	ST4	42
BHHS14	ST1	14
BHHS15	ST2	9
BHHS16	ST2	9
BHHS17	ST2	12
BHHS18	ST4	42
BHHS19	ST2	12
BHHS20	ST2	12
BHHS21	ST3	34
BHHS22	ST4	42
BHHS23	ST3	34
BHHS24	ST4	42
BHHS25	ST4	42
BHHS26	ST1	4
BHHS27	ST3	34
BHHS28	ST4	42
BHHS29	ST4	42
BHHS30	ST4	42
BHHS31	ST4	42
BHHS32	ST3	34
BHHS33	ST3	34
BHHS34	ST1	4
BHHS35	ST4	42
BHHS36	ST4	42
BHHS37	ST1	4
BHHS38	ST4	42
BHHS39	ST3	34
BHHS40	ST4	42
BHHS41	ST4	42
BHHS42	ST7	137
BHHS43	ST4	42
BHHS44	ST4	42
BHHS45	ST6	123
BHHS46	ST4	42

 https://doi.org/10.3390/pathogens14020139

BHHS47	ST1	4
BHHS48	ST4	42
BHHS49	ST4	42
BHHS50	ST4	42
BHHS51	ST1	4
BHHS52	ST4	42
BHHS53	ST3	34
BHHS54	ST3	34
BHHS55	ST1	4

Table S2. DATASET2, with haplotypes distribution; isolates from the present study are in bold

Isolate	Subtype	Allele	Haplotype	Region
JF274687	ST1	4	1	Lazio
KP284167	ST1	4	2	ND
**BHHS34**	ST1	4	5	Lazio
JF274662	ST1	4	5	Lazio
**BHSS55**	ST1	4	10	Lazio
**BHHS37**	ST1	4	8	Lazio
**BHHS14**	ST1	4	11	Lazio
**BHHS26**	ST1	4	9	Lazio
**BHHS47**	ST1	4	6	Lazio
**BHHS51**	ST1	4	7	Lazio
**BHHS3**	ST1	4	7	Lazio
**BHHS12**	ST1	4	7	Lazio
JF274667	ST1	75	3	Lazio
KP284168	ST1	77	4	ND
JF274676	ST2	70	12	Lazio
JF274693	ST2	70	12	Lazio
**BHHS6**	ST2	9	22	Lazio
JF274702	ST2	9	22	Sardinia
**BHHS2**	ST2	9	22	Lazio
KP284170	ST2	9	22	ND
**BHHS15**	ST2	9	13	Lazio
JF274659	ST2	12	20	Lazio
JF274664	ST2	12	20	Lazio
**BHHS19**	ST2	12	20	Lazio
**BHHS20**	ST2	12	20	Lazio
JF274671	ST2	12	14	Lazio
JF274672	ST2	12	15	Lazio
KP284169	ST2	12	16	ND
JF274701	ST2	61	21	Sardinia
JF274678	ST2	66	17	Lazio
JF274660	ST2	67	18	Lazio
JF274663	ST2	65	19	Lazio
JF274679	ST3	ND	23	Lazio
JF274666	ST3	128	24	Lazio
JF274688	ST3	128	24	Lazio
JF274668	ST3	128	25	Lazio

JF274700	ST3	36	39	Sardinia
**BHHS1**	ST3	36	39	Lazio
JF274682	ST3	36	35	Lazio
JF274686	ST3	34	29	Lazio
JF274698	ST3	34	31	Sardinia
KP284171	ST3	34	34	ND
**BHHS23**	ST3	34	34	Lazio
**BHHS4**	ST3	34	34	Lazio
**BHHS11**	ST3	34	34	Lazio
**BHHS27**	ST3	34	34	Lazio
**BHHS32**	ST3	34	34	Lazio
**BHHS33**	ST3	34	34	Lazio
**BHHS39**	ST3	34	34	Lazio
**BHHS53**	ST3	34	34	Lazio
**BHHS54**	ST3	34	34	Lazio
JF274658	ST3	34	34	Lazio
JF274661	ST3	34	34	Lazio
JF274670	ST3	34	34	Lazio
JF274675	ST3	34	34	Lazio
JF274681	ST3	34	34	Lazio
JF273694	ST3	34	34	Sardinia
JF274703	ST3	34	34	Sardinia
**BHHS21**	ST3	34	33	Lazio
JF274697	ST3	48	26	Sardinia
JF274674	ST3	37	32	Lazio
JF274683	ST3	46	36	Lazio
JF274685	ST3	47	37	Lazio
JF274699	ST3	49	38	Lazio
JF274669	ST3	53	27	Lazio
JF274680	ST3	54	28	Lazio
JF274696	ST3	55	30	Lazio
JF274689	ST4	89	40	Lazio
**BHHS31**	ST4	42	41	Lazio
**BHHS13**	ST4	42	41	Lazio
**BHHS7**	ST4	42	41	Lazio
**BHHS5**	ST4	42	41	Lazio
**BHHS9**	ST4	42	41	Lazio
**BHHS18**	ST4	42	41	Lazio
**BHHS24**	ST4	42	41	Lazio
**BHHS28**	ST4	42	41	Lazio
**BHHS29**	ST4	42	41	Lazio
**BHHS30**	ST4	42	41	Lazio
**BHHS8**	ST4	42	41	Lazio
**BHHS10**	ST4	42	41	Lazio
**BHHS22**	ST4	42	41	Lazio
**BHHS25**	ST4	42	41	Lazio
**BHHS36**	ST4	42	41	Lazio
**BHHS38**	ST4	42	41	Lazio
**BHHS40**	ST4	42	41	Lazio
**BHHS43**	ST4	42	41	Lazio
**BHHS35**	ST4	42	41	Lazio

**BHHS52**	ST4	42	41	Lazio
**BHHS41**	ST4	42	41	Lazio
JF274665	ST4	42	41	Lazio
JF274673	ST4	42	41	Lazio
JF274684	ST4	42	41	Lazio
JF274690	ST4	42	41	Lazio
JF274691	ST4	42	41	Lazio
KP284173	ST4	42	41	ND
**BHHS48**	ST4	42	41	Lazio
**BHHS44**	ST4	42	41	Lazio
**BHHS46**	ST4	42	41	Lazio
**BHHS49**	ST4	42	41	Lazio
**BHHS50**	ST4	42	41	Lazio
JF274692	ST8	21	42	Lazio
JF274677	ST8	95	43	Lazio
KP284175	ST8	95	43	ND
KP284174	ST6	123	44	ND
**BHHS45**	ST6	123	44	Lazio
**BHHS42**	ST7	137	45	Lazio
JF274695	ST7	111	46	Sardinia
